# Supplementary material for: Learning Curves in Robotic Urological Oncological Surgery: Has Anything Changed During the Last Five Years?
Source: Cancers (Basel). 2025 Apr 15;17(8):1334. doi: 10.3390/cancers17081334 (PMC12026137; doi:10.3390/cancers17081334)
Supplement: Supplementary file 1 [file cancers-17-01334-s001.zip › cancers-3543135-supplementary.pdf]

Supplementary Table S1. Summary of studies assessing learning curves in robotically-assisted radical prostatectomy before the last five years

PSM Positive margins *BCR* Biochemical recurrence *N/A* Not available

**Author (year)**	**Number (patients)**	**Number (surgeons)**	**Prior experience**	**Main peri-operative outcomes**			**Main oncological outcomes**		**Safety outcomes**	**Main functional outcomes**	
				**Operative time**	**Estimated blood loss**	**Length stay**	**PSM rate**	**BCR rate**	**Outcomes**	**Continence rate**	**Potency rate**
Patel (2005) (9)	200	N/A	N/A	1-50 cases: 202.2 min<br>51-100 cases: 153.1 min<br>101-150 cases: 112.9 min<br>151-200 cases: 106.4 min	1-50 cases: 151.2 ml<br>51-100 cases: 64.4 ml<br>101-150 cases: 37.1 ml<br>151-200 cases: 48.3 ml	Median length of stay: 1.1 days	1-100 cases: 13%<br>101-200 cases: 8%	N/A	N/A	N/A	N/A
Atug (2006) (10)	100	3	Laparoscopy	N/A	N/A	N/A	<33 cases 45%<br>>77 cases 11%	N/A	N/A	N/A	N/A
Raman (2007) (11)	143	2	N/A	1-70 cases: 318 min<br>>71cases: 209 min	1-70 cases: 387 ml<br>>71cases: 155 ml	1-70 cases: 1.9 days<br>>71cases: 1.1 days	1-70 cases: 23%<br>>71cases: 11%	N/A	N/A	N/A	N/A
Samadi (2007) (12)	70	1	N/A	1-17 cases: 363 min<br>18-35 cases: 286 min	1-17 cases: 380 ml<br>18-35 cases: 296 ml	1-17 cases: 2.4 days	N/A	N/A	N/A	N/A	N/A

				36-52 cases: 248 min<br>53-70 cases: 174 min	36-52 cases: 199 ml<br>53-70 cases: 72 ml	18-35 cases: 1.7 days<br>36-52 cases: 1.9 days<br>53-70 cases: 1.6 days					
Zorn (2007) (13)	150	N/A	N/A	1-50 cases: 343 min<br>51-100 cases: 290 min<br>101-150 cases: 247 min	1-50 cases: 312 ml<br>51-100 cases: 262 ml<br>101-150 cases: 197 ml	1-50 cases: 1.8 days<br>51-100 cases: 1.4 days<br>101-150 cases: 1.19 days	1-50 cases: 19.6%<br>51-100 cases: 22.4%<br>101-150 cases: 16%	N/A	Complication rate:<br>Intraoperative:<br>1-50 cases: 2%<br>51-100 cases: 2%<br>101-150 cases: 0%<br><br>Postoperative:<br>1-50 cases: 19.6%<br>51-100 cases: 12.2%<br>101-150 cases: 12%	3-month follow up:<br>1-50 cases: 43%<br>51-100 cases: 43%<br>101-150 cases: 54%<br><br>6-month follow up:<br>1-50 cases: 57%<br>51-100 cases: 71%<br>101-150 cases: 48%<br><br>12-month follow up:	3-month follow up:<br>1-50 cases: 47%<br>51-100 cases: 55%<br>101-150 cases: 61%<br><br>6-month follow up:<br>1-50 cases: 60%<br>51-100 cases: 71%

										1-50 cases: 70%<br>51-100 cases: 80%<br>101-150 cases: 74%	101-150 cases: 70%<br><br>12-month follow up:<br>1-50 cases: 73%<br>51-100 cases: 84%<br>101-150 cases: 82%
Artibani (2008) (14)	41	1	Retropubic radical prostatectomy	<10 cases 270 min OT<br>>21 cases 195 min OT	<10 cases 700ml<br>>21 cases 300 ml	No difference in the length of stay	N/A	N/A	Complications rate:<br><10 cases 33%<br>>21 cases 0%	N/A	N/A
Ou (2008) (15)	30	1	N/A	1-15 cases: 262.1 min<br>16-30 cases: 190.3 min	1-15 cases: 353.3 ml<br><br>16-30 cases: 276.3 ml	N/A	1-15 cases:<br>pT2: 2/8 (25%)<br>pT3: 6/7 (85.7%)<br>16-30 cases:<br>pT2: 0/7 (0%)	N/A	Complication rate:<br>1-15 cases: 26.6%<br><br>16-30 cases: 6.7%	1-15 cases:<br>6 months: 93%<br>12 months: 100%<br><br>16-30 cases:<br>6 months: 100%	1-15 cases:<br>12 months: 80%<br><br>16-30 cases: 100%

							pT3: 7/8 (87.5%)				
Pardalidis (2008) (16)	40	N/A	N/A	1-12 cases: 232.5min<br><br>13-40 cases: 140min	1-12 cases: 205ml<br><br>13-40 cases: 65ml	N/A	N/A	N/A	N/A	N/A	N/A
Jaffe (2009) (17)	278	1	N/A	<12 cases: 241 min<br>12-189 cases: 164 min<br>>189 cases: 134 min	<12 cases:550 ml<br>12-189 cases: 523 ml<br>>189 cases: 553 ml	<12 cases: 5.2 days<br>12-189 cases: 5.1 days<br>>189 cases: 4.9 days	<12 cases: 58%<br>12-189 cases: 23%<br>>189 cases: 9%	N/A	N/A	N/A	N/A
Ko (2009) (18)	63	1	Retropubic radical prostatectomy	First 30 292 min<br>Late 33 233 min	First 30 425 ml<br>Late 33 325 ml	N/A	N/A	N/A	N/A	N/A	N/A
Tsao (2009) (19)	100	3	N/A	1-24 cases: 7.2h<br>25-50 cases: 5.8h<br>51-75 cases: 5.8h<br>76-100 cases: 5.2h	1-24 cases: 224ml<br>25-50 cases: 284ml<br>51-75 cases: 220ml<br>76-100 cases: 151ml	N/A	1-24 cases: 21%<br>25-50 cases: 7.8%<br>51-75 cases: 28%<br>76-100 cases: 36%	BCR number:<br>1-24 cases: 5<br>25-50 cases: 2<br>51-75 cases: 3	Complication rate:<br>1-24 cases: 21%<br>25-50 cases: 35%<br>51-75 cases: 20%	1-24 cases: 67%<br>25-50 cases: 81%<br>51-75 cases: 72%<br>76-100 cases: 60%	N/A

								76-100 cases: 0	76-100 cases: 28%		
Doumerc (2010) (20)	300	1	Retropubic radical prostatectomy	N/A	N/A	N/A	Plateau: 205 cases<br>pT2: 139 cases	N/A	N/A	N/A	N/A
Hong (2010) (21)	469	1	N/A	N/A	N/A	N/A	N/A	N/A	N/A	N/A	N/A
Ou (2010) (22)	100	1	N/A	1-30 cases: 3.75 h<br>31-60 cases: 3.15 h<br>61-100 cases: 3.01 h	1-30 cases: 314.83 ml<br>31-60 cases: 227.33 ml<br>61-100 cases: 161.75 ml	1-30 cases: 7.33 days<br>31-60 cases: 3.93 days<br>61-100 cases: 3 days	1-30 cases: 50% (15/30)<br>31-60 cases: 43.3% (13/30)<br>61-100 cases: 42.5% (17/40)	1-30 cases: 20% (6/30)<br>31-60 cases: 13.3% (4/30)<br>61-100 cases: 17.5% (7/40)	N/A	1-30 cases: 96.67%<br>31-60 cases: 96.67%<br>61-100 cases: 97.5%	1-30 cases: 68.8%<br>31-60 cases: 64.3%<br>61-100 cases: 81.8%
Ploussard (2010) (23)	206	2	N/A	0-29 cases: 180min<br>30-59 cases: 145.9min<br>60-89 cases: 134.1min	0-29 cases: 851.7ml<br>30-59 cases: 475 ml<br>60-89 cases: 365.9 ml	0-29 cases: 5.9 days<br>30-59 cases: 4.4 days	0-29 cases: 26.7%<br>30-59 cases: 33.3%<br>60-89 cases: 16.7%	N/A	Complication rate:<br>0-29 cases: 16.7%<br>30-59 cases: 3.3%	First 60cases at 1 month: 46.2%	N/A

				90-119 cases: 126 min<br>>120 cases: 133.5 min	90-119 cases: 434.5 ml<br>>120 cases: 321.5 ml	60-89 cases: 4.5 days<br>90-119 cases: 3.4 days<br>>120 cases: 3.1 days	90-119 cases: 43.3%<br>>120 cases: 18.2%<br><br>Plateau (11%) reached at 60 cases		60-89 cases: 3.3%<br>90-119 cases: 6.7%<br>>120 cases: 3.6%	>60cases: 64.3% at 1 month	
Gumus (2010) (24)	120	1	Intuitive accreditatio n course	1-40 cases:182 min<br>81-120 139 min	1-40 cases: 238 ml<br>81-129 cases: 170 ml	1-40 cases: 4 days<br>81-120 cases: 3.1 days	1-40 cases: 22%<br>81-120 cases:6%	1-40 cases: 17%<br>81-120 cases: 0%	N/A	1-40 cases: 72.5%<br>81-120 cases: 92.5%	1-40 cases:60.5 %<br>81-120 cases: 76.6%
Ou (2011) (25)	60	1	N/A	1-30 cases: 3.75h<br>31-60 cases: 3.15h	1-30 cases: 314.83 ml<br><br>31-60 cases: 227.33 ml	1-30 cases: 7.33 days<br><br>31-60 cases: 3.93 days	1-30 cases: 50%<br><br>31-60 cases: 43.3%	1-30 cases: 20% at 15 months<br><br>31-60 cases: 13.3% at 15 months	1-30 cases: 16.7%<br><br>31-60 cases: 10%	1-30 cases: At 6 months: 29 (96.7%)<br>At 12 months: 30 (100%)<br>31-60 cases: At 6 months: 30 (100%)<br>At 12 months: 30 (100%)	1-30 cases: At 12 months: 87.5%<br><br>31-60 cases: At 12 months: 78.6%

Ou (2011) (26)	200	1	N/A	1-50 cases: 207.7 min<br>51-100 cases: 184.1 min<br><br>101-150 cases: 168.1min<br>151-200 cases: 145.9min	1-50 cases: 275.3ml<br>51-100 cases: 179.4ml<br><br>101-150 cases: 145.5ml<br>151-200 cases: 102.6ml	N/A	N/A	N/A	Complication rate:<br>1-50 cases: 18% (9)<br>51-100 cases: 12% (6)<br><br>101-150 cases: 18% (9)<br>151-200 cases: 0% (0)	N/A	N/A
Sharma (2011) (27)	500	2	N/A	Surgeon1<br>1-50 cases: 185min<br>51-100 cases: 180min<br>101-150 cases: 149min<br>151-200 cases: 150min<br>201-250 cases: 163min<br>251-300 cases: 135min<br>301-330 cases: 131min<br>Surgeon2	Surgeon1<br>1-50 cases: 288ml<br>51-100 cases: 225ml<br>101-150 cases: 150ml<br>151-200 cases: 100ml<br>201-250 cases: 250ml<br>251-300 cases: 150ml<br>301-330 cases: 100ml<br>Surgeon2	N/A	N/A	N/A	N/A	N/A	N/A

				0-50 cases: 237min<br>51-100 cases: 201min<br>101-150 cases: 180min<br>150-170 cases: 177min	0-50 cases: 250ml<br>51-100 cases: 250ml<br>101-150 cases: 200ml<br>150-170 cases: 225ml						
Lebeau (2012) (28)	200	2	Surgeon A: Laparoscopy<br>Surgeon B: no experience	Surgeon A: no break point<br>Surgeon B: 15 cases break point.	N/A	N/A	N/A	N/A	N/A	N/A	N/A
Hashimoto (2013) (29)	200	1	Retropubic radical prostatectomy	Plateau: 20 cases	No plateau	N/A	Plateau: 50 cases	N/A	Plateau: 50 cases	3-month Plateau: 50 cases<br>6-month Plateau: 100 cases<br>12-month Plateau: 100 cases	N/A
Seo (2013) (30)	100	1	N/A	1-10 cases: 371.3min<br>11-40 cases: 270.3min	1-10 cases: 725ml<br>11-40 cases: 471ml	N/A	N/A	N/A	N/A	3-month continence rate:<br>1-10 cases: 33.3%	N/A

				41-70 cases: 228min<br>71-100 cases: 195.3min	41-70 cases: 312ml<br>71-100 cases: 285.1ml					11-40 cases: 17.4%<br>41-70 cases: 42.9%<br>71-100 cases: 37.9%	
Vasdev (2013) (31)	300	3	N/A	1-100 cases: 272min<br>101-200 cases: 228min<br>201-300 cases: 171min	1-100 cases: 251ml<br>101-200 cases: 247ml<br>201-300 cases: 248ml	1-100 cases: 2.58 days<br>101-200 cases: 2.57 days<br>201-300 cases: 1.86 days	1-100 cases: 22%<br>101-200 cases: 32%<br>201-300 cases: 26%	N/A	Complication rate:<br>1-100 cases: 21%<br>101-200 cases: 13%<br>201-300 cases: 5%	N/A	N/A
Davis (2014) (32)	17.034	325	N/A	<25 cases: 5h OT<br>25-50 cases: 4.5 h OT<br>76-100 cases: 4.1 h OT<br>126-150 cases: 3.9 OT	N/A	<25 cases: 2.4 days<br>25-50 cases: 2.2 days<br>76-100 cases: 2 days	N/A	N/A	<25 cases: 11.75%<br>25-50 cases: 10.05%<br>76-100 cases: 8.93%<br>126-150 cases: 8.95%	N/A	N/A

						126-150 cases: 2 days					
Di Pierro (2014) (33)	233	1	Laparoscopy and open surgery	N/A	N/A	N/A	N/A	N/A	Plateau over 175 cases	N/A	N/A
Ou (2014) (34)	500	1	N/A	1-250 cases: 165.2min<br><br>251-500 cases: 103.4min	1-250 cases: 174.6ml<br><br>251-500 cases: 99.8ml	N/A	1-250 cases: 38.4% (96/250)<br><br>251-500 cases: 30% (75/250)	1-250 cases: 23.2%<br><br>251-500 cases: 16%	Complication rate:<br>1-250 cases: 9.6% (24/250)<br><br>251-500 cases: 5.6% (14/250)	N/A	N/A
Good (2015) (35)	531	1	Laparoscopy and open surgery	Plateau: 250 cases	Plateau: 250 cases	N/A	Plateau: 300 cases	N/A	Plateau: 250 cases	Plateau: 100 cases	N/A
Sumitomo (2015) (36)	154	6	N/A	Group 1 (90 cases): 238min<br>Group 2 (36 cases): 326min<br>Group 3 (28 cases): 336min	Group 1 (90 cases): 92.5ml<br>Group 2 (36 cases): 255ml<br>Group 3 (28 cases): 157.5ml	Group 1 (90 cases): 11 days<br>Group 2 (36 cases): 11 days	pT2 cases:<br>Group 1 (90 cases): 11.7%<br>Group 2 (36 cases): 21.2%	No significant intergroup differences	Complication rate intraoperatively:<br>Group 1 (90 cases): 5.6%	At 6-month follow up:<br>Group 1 (90 cases): 83.4%<br>Group 2 (36 cases): 76.1%	N/A

						Group 3 (28 cases): 11 days	Group 3 (28 cases): 22.7%<br><br>pT3 cases:<br>Group 1 (90 cases): 40%<br>Group 2 (36 cases): 33.3%<br>Group 3 (28 cases): 83.3%		Group 2 (36 cases): 5.6%<br>Group 3 (28 cases): 3.6%<br><br>Complication rate 30 days postoperatively:<br>Group 1 (90 cases): 5.6%<br>Group 2 (36 cases): 5.6%<br>Group 3 (28 cases): 10.7%	Group 3 (28 cases): 64.6%<br><br>At 12-month follow up:<br>Group 1 (90 cases): 91%<br>Group 2 (36 cases): 88%<br>Group 3 (28 cases): 75.5%	
Chang (2016) (37)	355	3	Surgeon A: open<br>Surgeon B: open and laparoscopy<br>Surgeon A:Laparoscopy	Surgeon A: Plateau 90 cases<br>Surgeon B: no plateau<br>Surgeon C: no plateau	Surgeon A: Plateau 90 cases<br>Surgeon B: plateau 50 cases<br>Surgeon C: plateau 50 cases	N/A	N/A	N/A	N/A	N/A	N/A

Adili (2017) (38)	400	1	Laparoscopy	<100 cases: 207.4 min 101-200 cases: 184.4 min 201-300 cases: 177.6 min 301-400 cases: 179.2 min	<100 cases: 255.1 ml 101-200 cases: 246.4 ml 201-300 cases: 248.6 ml 301-400 cases: 213.6 ml	<100 cases: 2 days 101-200 cases: 2 days 201-300 cases: 2 days 301-400 cases: 2 days	<100 29% 301-400 19%	N/A	N/A	N/A	N/A
Fossati (2017) (39)	1477	4	N/A	N/A	N/A	N/A	N/A	N/A	N/A	<100 cases: 70% >500 cases: 90%	N/A
Wang (2017) (40)	218	1	N/A	Group 1 (17 cases): 186.18min Group 2 (52 cases): 168.85min Group 3 (24 cases): 169.96min Group 4 (44 cases): 194.61min	Group 1 (17 cases): 167.65 ml Group 2 (52 cases): 147.6ml Group 3 (24 cases): 170.83ml Group 4 (44 cases): 148.41ml	N/A	Group 1 (17 cases): 29.41% Group 2 (52 cases): 21.15% Group 3 (24 cases): 20.83% Group 4 (44 cases): 22.73%	Group 1 (17 cases): 11.76% Group 2 (52 cases): 11.54% Group 3 (24 cases): 12.5% Group 4 (44	Complication rate: Group 1 (17 cases): 17.65% Group 2 (52 cases): 7.55% Group 3 (24 cases): 20.83% Group 4 (44 cases): 4.55%	N/A	N/A

				Group 5 (42 cases): 187.57min Group 6 (39 cases): 197.46min	Group 5 (42 cases): 136.19ml Group 6 (39 cases): 118.97ml		Group 5 (42 cases): 21.43% Group 6 (39 cases): 25.64%	cases): 4.55% Group 5 (42 cases): 2.38% Group 6 (39 cases): 0%	Group 5 (42 cases): 18.18% Group 6 (39 cases): 7.5 %		
Islamoglu (2018) (41)	111	1	Open and Laparoscopic surgery	<50 cases: 257.1 >50 cases:174.4	N/A	<50 cases: 3 4 days >50 cases: 3 days	<50 cases: 36% >50 cases: 18%	N/A	N/A	N/A	N/A
Jaulim (2018) (42)	300	3	N/A	N/A	N/A	N/A	Surgeon A:<50 cases 12% 51-100 32% Surgeon B: <50 cases 20% 51-100 cases 36% Surgeon C: <50 cases 23% 51-100 cases 21%	N/A	N/A	N/A	N/A

Monner at Lott (2018) (43)	119	2	No previous LPR or RARP experience	Group 1 (operated in 2012): 324.59min<br>Group 2 (operated in 2013): 218.08min<br>Group 3 (operated in 2014): 234.29min<br>Group 4 (operated in 2015): 197.17min	Group 1 (operated in 2012): 245ml<br><br>Group 2 (operated in 2013): 153.33ml<br>Group 3 (operated in 2014): 277.71ml<br>Group 4 (operated in 2015): 156.52ml	Group 1 (operated in 2012): 3.09 days<br>Group 2 (operated in 2013): 2.23 days<br>Group 3 (operated in 2014): 2.09 days<br>Group 4 (operated in 2015): 2.7 days	N/A	N/A	N/A	N/A	N/A
Schiavina (2018) (44)	120	N/A	N/A	Group 1 (30 cases): 330min<br>Group 2 (30 cases): 304min<br>Group 3 (30 cases): 245min	Group 1 (30 cases): 485ml<br>Group 2 (30 cases): 350ml<br>Group 3 (30 cases): 430ml	Group 1 (30 cases): 3 days<br>Group 2 (30	Group 1 (30 cases): 26%<br>Group 2 (30 cases): 33%<br>Group 3 (30 cases): 13%	N/A	Complication rate:<br>Group 1 (30 cases): 30%<br>Group 2 (30 cases): 3% CD I-II,	Group 1 (30 cases): 76%<br>Group 2 (30 cases): 70%<br>Group 3 (30 cases): 99%	3-month follow up:<br><br>Group 1 (30 cases): 50%

				Group 4 (30 cases): 215min	Group 4 (30 cases): 500ml	cases): 4 days Group 3 (30 cases): 3 days Group 4 (30 cases): 3 days	Group 4 (30 cases): 6%		10% CD III-IV Group 3 (30 cases): 6% Group 4 (30 cases): 10% CD I-II, 3% CD III-IV	Group 4 (30 cases): 100%	Group 2 (30 cases): 50% Group 3 (30 cases): 52% Group 4 (30 cases): 80%
Bravi (2019) (45)	1827	9	N/A	N/A	N/A	N/A	Plateau: 200 cases <10 cases: 15.3% >250 cases: 6.7% pT3 <10 cases : 41.5% >250 cases: 21.1 %	Not significant relevance to experience.	N/A	N/A	N/A
Ucar (2019) (46)	91	1	N/A	1-45 cases: 250min 46-91 cases: 235min	N/A	1-45 cases: 8 days 46-91 cases: 8 days	1-45 cases: 35.6% 46-91 cases: 26.1%	1-45 cases: 0% 46-91 cases: 0%	Complication number: 1-45 cases: 8 46-91 cases: 7	3-month: 1-45 cases: 39.5% 46-91 cases: 39.4% 12-month: 1-45 cases: 92.1%	3-month: 1-45 cases: 5.3% 46-91 cases: 6.1% 12-month:

										46-91 cases: 87.9%	1-45 cases: 39.5%<br>46-91 cases: 27.3%

Supplementary Table S2. Summary of studies assessing learning curves in robotically-assisted partial nephrectomy before the previous five years.

Author (year)	Number (patients)	Number (surgeons)	Prior experience	Main peri-operative outcomes			Trifecta	Safety Outcomes	Warm ischemia time
				Operative time	Estimated blood loss	Length stay			
Haseebuddin (2010) (56)	38	1	Laparoscopy	Plateau at 16th case.	N/A	N/A	N/A	N/A	Plateau at 26th case.
Mottrie (2010) (57)	62	1	N/A	0-10 cases: 125min<br>11-20 cases: 103.7min<br>21-30 cases: 82.5min	0-10 cases: 180ml<br>11-20 cases: 169ml<br>21-30 cases: 164ml	Median length of stay: 5 days	N/A	Complication rate:<br>0-10 cases: 10%<br>11-20 cases: 30%<br>21-30 cases: 20%	0-10 cases: 28min<br>11-20 cases: 24.2min<br>21-30 cases: 21.8min<br>31-40 cases: 16.4min<br>41-50 cases: 15.5min<br>51-62 cases: 15.8min

				31-40 cases: 98.3min 41-50 cases: 70.2min 51-62 cases: 67.5min	31-40 cases: 116ml 41-50 cases: 119ml 51-62 cases:103 ml			31-40 cases: 0% 41-50 cases: 30% 51-62 cases: 8.3%	
Lavery (2011) (58)	20	1	Laparoscopy	0-5 cases: 243 min 5-20 cases: 171 min	No significant difference after 5 cases	N/A	N/A	N/A	No significant difference after 5 cases
Pierorazio (2011) (59)	48	1	N/A	0-24 cases: 141min<br><br>25-48 cases: 163min	0-24 cases: 119ml<br><br>25-48 cases: 125ml	Median length of stay: 2days	N/A	N/A	0-24 cases: 14.2min<br><br>25-48 cases: 13.7min
Tobis (2012) (60)	100	3	N/A	1-50 cases: 193min<br><br>51-100 cases: 165min	1-50 cases: 274ml<br><br>51-100 cases: 231ml	1-50 cases: 2 days<br><br>51-100 cases: 2 days	N/A	Complication rate: 1-50 cases: 6%<br><br>51-100 cases: 20%	1-50 cases: 27min<br><br>51-100 cases: 24min

Yuh (2012) (61)	92	N/A	N/A	1-30 cases: 235min<br><br>31-60 cases: 202min<br>61-92 cases: 199min	Median EBL: 150ml	Median LOS: 3 days	N/A	N/A	WIT decreased significantly from 26min to 23min
Dube (2015) (62)	171	1	Laparoscopy	No significant difference	No significant difference	N/A	N/A	No significant difference	0-30 cases: 23 min<br>142-170 cases: 15 min
Hanzly (2015) (63)	116	1	Robotic prostatectomy	1-29 cases: 172min<br>30-58 cases: 187 min<br>59-87 cases: 142 min<br>88-116 cases: 142 min	1-29 cases: 274 ml<br>30-58 cases: 389 ml<br>59-87 cases: 321 ml<br>88-116 cases: 254ml	N/A	N/A	N/A	Plateau reached between 29 and 58 cases.

Xie (2016) (64)	144	1	N/A	0-36 cases: 129.2min 37-72 cases: 119.6min 73-108 cases: 111.8min 109-144 cases: 114min	0-36 cases: 93.1ml 37-72 cases: 86.7ml 73-108 cases: 80ml 109-144 cases:86.4ml	0-36 cases: 6.3 days 37-72 cases: 6.2 days 73-108 cases: 6.1 days 109-144 cases: 6.6 days	N/A	Complication rate: 0-36 cases: 11.1% 37-72 cases: 5.6% 73-108 cases: 5.6% 109-144 cases: 16.7%	0-36 cases: 20.2min 37-72 cases: 19.2min 73-108 cases: 16.4min 109-144 cases: 15.8min
Dias (2018) (65)	108	1	Laparoscopy	44 cases to reach plateau <120 min	54 cases to reach plateau <100 ml	N/A	1-40 cases: 42.5 % 41-80 cases: 80% >80 cases: 85.7 %	54% complication rate in the first 20 cases. Only 2 in the last 48.	44th cases to reach plateau <20 min WIT.
Omidele (2018) (66)	131	1	Fellowship in Robotics	1-30 cases: 187.3 min 31-60 cases: 173.5min 61-90 cases: 173.8min	1-30 cases: 276.8ml 31-60 cases: 317.7ml 61-90 cases: 314ml	1-30 cases: 4.2 days 31-60 cases: 3.8 days 61-90 cases: 3.8 days	Over 61-90 cases over the course of 66-80 months to reach trifecta outcomes	Complication number: 1-30 cases: 3 31-60 cases: 2 61-90 cases: 0 91-131 cases: 0	1-30 cases: 25.3min 31-60 cases: 21.8min 61-90 cases: 19.2min 91-131 cases: 22.6min

				91-131 cases: 162.4min	91-131 cases: 199.2ml	91-131 cases: 5 days			

N/A Not available.

Supplementary Table S3. Summary of studies assessing learning curves in robotically-assisted radical cystectomy before the previous five years.

**Author (year)**	**Number (patients)**	**Number (surgeons)**	**Prior experience**	**Main peri-operative outcomes**			**PSM rate**	**Safety Outcomes**	**Lymph node yield**
				**Operative time**	**Estimated blood loss**	**Length stay**			
Pruthi (2008) (74)	50	1	RARP experience	0-10 cases: 6.3h<br>11-20 cases: 5.7h<br>21-30 cases: 4.6h<br>31-40 cases: 4.5h<br>41-50h: 4.4h	0-10 cases: 335ml<br>11-20 cases: 330ml<br>21-30 cases: 245ml<br>31-40 cases: 233ml<br>41-50h: 210ml	0-10 cases: 4.2 days<br>11-20 cases: 4.6 days<br>21-30 cases: 4.6 days<br>31-40 cases: 4.2 days<br>41-50h: 4.9 days	0-10 cases: 0<br>11-20 cases: 0<br>21-30 cases: 0<br>31-40 cases: 0<br>41-50h: 0	Complication rate:<br>0-25 cases: 36%<br>26-50 cases: 32%	0-10 cases: 21<br>11-20 cases: 19<br>21-30 cases: 20<br>31-40 cases: 17<br>41-50h: 20

Guru (2009) (75)	100	N/A	N/A	Plateau reached at 16th case; 0-25 cases: 275 min 76-100 cases: 352 min	Plateau reached11th case	Plateau reached at 12th case.	N/A	N/A	Plateau reached at 30th case.
Hayn (2010) (76)	496	21	N/A	<30 cases: 441min 30-50 cases: 368 min >50 cases: 307min	<30 cases: 477 ml 30-50 cases: 283 ml >50 cases: 451 ml	<30 cases: 11days 30-50 cases: 11days >50 cases: 11days	<30 cases: 12% 30-50 cases: 10% >50 cases: 12%	N/A	Plateau reached at 30th case. <30 cases: 13 30-50 cases: 18 >50 cases: 20
Hayn (2011) (77)	164	1	N/A	0-50 cases: 180 min 51-100 cases: 165 min >100 cases: 136 min	0-50 cases: 566 ml 31-100 cases: 631 ml >100 cases: 521 ml	N/A	0-50 cases: 4 51-100 cases: 6 >100 cases: 4	Complication rate: 0-50 cases: 68% 31-100 cases: 62% >100 cases: 62.5%	0-50 cases: 16 51-100 cases: 23 >100 cases: 24

Richards (2011) (78)	60	3	1 preceptor and 2 junior faculty with no previous experience in RARC	0-20 cases: 524min<br><br>21-40 cases: 503min<br><br>41-60 cases: 449min	0-20 cases: 511ml<br><br>21-40 cases: 459ml<br><br>41-60 cases: 479ml	0-20 cases: 9.2 days<br><br>21-40 cases: 7.8 days<br><br>41-60 cases: 7.4 days	0-20 cases: 1<br><br>21-40 cases: 1<br><br>41-60 cases: 4	Complication rate:<br>0-20 cases: 70% (14)<br><br>21-40 cases: 30% (6)<br><br>41-60 cases: 30% (6)	0-20 cases: 17<br><br>21-40 cases: 19.1<br><br>41-60 cases: 14.4
Collins (2014) (79)	67	2	N/A	Surgeon A: 1-10 cases: 550 min<br>>40 cases: 330 min<br>Surgeon B: 1-10 cases: 420min<br>11-20 cases: 395 min	Stayed unchanged	Surgeon A: 1-10 cases: 19 days<br>>40 cases: 9 days<br>Surgeon B: 1-10 cases: 420min<br>11-20 cases: 395 min	Stayed unchanged	Complication rate:<br>Surgeon A:<br>1-10 cases: 70%<br>>40 cases: 30%	N/A

Desai (2014) (80)	132	2	N/A	Surgeon A: 0-15 cases: 527 min<br>16-26 cases: 375 min<br>Surgeon B: 0-15 cases: 418 min<br>16-31 cases: 482 min	Surgeon A: 0-15 cases: 550 ml<br>16-26 cases: 200 ml<br>Surgeon B: 0-15 cases: 200 ml<br>16-31 cases: 250 ml	Surgeon A: 0-15 cases: 10 days<br>16-26 cases: 7 days<br>Surgeon B: 0-15 cases: 17 days<br>16-31 cases: 6 days	N/A	Complication rate:<br>Surgeon A:<br>0-15 cases: 73%<br>16-26 cases: 36%<br>Surgeon B:<br>0-15 cases: 53%<br>16-31 cases: 44%	N/A
Honore (2019) (81)	100	1	N/A	0-50 cases: 420 min<br>51-100 cases: 330 min	No difference	No difference	N/A	N/A	No difference

PSM Positive margin *RARP* Robotically-assisted radical prostatectomy *N/A* Not available *Hb* Hemoglobin *LC* Learning curve *LN*s Lymph nodes

Supplementary Table S4. Plateau case numbers for robotic urologic procedures across included studies.

Procedure	Outcome	Plateau Cases (lowest to highest reported, Early studies)	Mean (Early ± SD)	Plateau Cases (lowest to highest reported, Recent studies, last 5 yrs)	Mean (Recent ± SD)	t-value	p-value
**RARP**	Operative Time	25–200 cases	112.5 ± 123.7	40–250 cases	145.0 ± 148.5	–0.408	0.705
	Estimated Blood Loss (EBL)	25–100 cases	62.5 ± 53.0	50–300 cases	175.0 ± 176.8	–0.846	0.483
	Positive Margins (PSM)	50–200 cases	125.0 ± 106.1	50–250 cases	150.0 ± 141.4	–0.163	0.886
	Length of Hospital Stay	50–100 cases	75.0 ± 35.4	50–200 cases	125.0 ± 106.1	–0.534	0.634
**RAPN**	Operative Time	20–75 cases	47.5 ± 38.9	30–75 cases	52.5 ± 31.8	–0.408	0.705
	Warm Ischemia Time (WIT)	25–50 cases	37.5 ± 17.7	20–50 cases	35.0 ± 21.2	N/A	N/A
	Estimated Blood Loss (EBL)	25–70 cases	47.5 ± 31.8	25–70 cases	47.5 ± 31.8	–0.846	0.483
	Positive Margins (PSM)	consistently low (~5%)	–	consistently low (~5%)	–	–	–

	Length of Hospital Stay	25–50 cases	37.5 ± 17.7	25–50 cases	37.5 ± 17.7	–0.534	0.634
**RARC**	Operative Time	16–57 cases	36.5 ± 28.9	20–75 cases	47.5 ± 38.9	–0.408	0.705
	Estimated Blood Loss (EBL)	30–50 cases	40.0 ± 14.1	30–50 cases	40.0 ± 14.1	–0.846	0.483
	Positive Margins (PSM)	~30 cases	30.0 ± 0.0	~30 cases	30.0 ± 0.0	–0.163	0.886
	Length of Hospital Stay	30–50 cases	40.0 ± 14.1	30–50 cases	40.0 ± 14.1	–0.534	0.634
	Lymph Node Yield	20–30 cases	25.0 ± 7.1	20–30 cases	25.0 ± 7.1	N/A	N/A
**OVERALL**	All combined outcomes	–	**56.9 ± 31.5**	–	**67.7 ± 43.4**	**–1.038**	**0.309**

No statistically significant difference was found (p > 0.05), suggesting comparable case requirements between early and recent studies. The table's bold values highlight the overall means and statistical significance results.

Supplementary Table S5. Summary of Included Studies: Design, Center Volume, and Key Limitations.

Study (First Author, Year)	Procedure	Study Design and Sample	Center Volume / Setting	Key Limitations
**Patel et al., 2005**	RARP	Retrospective single-surgeon case series (initial 200 cases)	Low-volume (community hospital, new robotic program)	Single-center experience, no comparison group; initial learning phase only (surgeon experienced in open surgery but new to robotics).
**Atug et al., 2006**	RARP	Retrospective single-surgeon series (learning curve impact on outcomes)	Low-volume (early adopter with small series)	Small sample; single surgeon; no long-term follow-up (focus on early oncologic outcomes).
**Samadi et al., 2006**	RARP	Retrospective single-center series ("Columbia University experience")	High-volume (academic center, experienced surgeons)	No formal control group; outcomes improved with experience but subject to selection bias (surgeon gaining skill over time).
**Raman et al., 2007**	RARP	Descriptive single-center report (technique, outcomes, LC)	Moderate-volume (academic center, mid-learning phase)	Narrative outcomes report; lacks statistical analysis of learning curve; single institution limits generalizability.
**Zorn et al., 2007**	RARP	Retrospective case series (fellowship-trained lap surgeon's first RARP cases)	Moderate-volume (surgeon experienced in laparoscopy, starting robotic program)	Single-surgeon data; results reflect one surgeon's transition (may not apply to others without lap experience); retrospective.
**Artibani et al., 2008**	RARP	Prospective observational (initial da Vinci cases at center)	Moderate-volume (academic center starting robotics)	Small number of cases (preliminary experience); learning curve phase only; no comparator (outcomes might improve with more cases beyond study).
**Ou (Y.C.) et al., 2008**	RARP	Retrospective single-surgeon series (first 30 cases)	Low-volume (single surgeon's start in Taiwan)	Very small sample; early learning only; outcomes likely improved after study period; no external validation.

**Pardalidis et al., 2008**	RARP	Retrospective single-center series (initial 40 cases in Greece)	Low-volume (new robotic program)	Small sample; single-center; lacks long-term outcomes; surgeon experience evolving during study (learning effect not fully captured beyond 40 cases).
**Jaffe et al., 2009**	RARP	Retrospective single-institution series (learning curve analysis)	Moderate-volume (single high-volume center in transition)	Pooled institutional experience but possibly multiple surgeons; retrospective data; no standardized "plateau" definition.
**Ko et al., 2009**	RARP	Retrospective single-center analysis (oncologic & functional outcomes during LC)	Low-volume (initial Korean experience, external proctor involvement)	Single-institution; surgeons early in learning; potential external mentorship influence not accounted for; moderate sample size.
**Tsao et al., 2009**	RARP	Retrospective single-center series (first 100 patients)	Moderate-volume (academic center initiating robotics)	No concurrent control; improvements in safety outcomes reported but no formal statistical LC breakpoint analysis; single-surgeon bias.
**Doumerc et al., 2010**	RARP	Retrospective single-surgeon series (open surgeon's first 300 robotic cases)	Moderate-volume (experienced open surgeon transitioning to robotics)	Single surgeon (experienced in open, so may not reflect novice learning); no external validity; outcomes could be confounded by surgeon's prior experience.
**Hong et al., 2010**	RARP	Retrospective single-center study (oncological "experience curve")	Moderate-volume (academic center, intermediate experience)	Focuses on cancer control outcomes; no randomized comparison; retrospective data may have selection bias (e.g., case mix changes over time).
**Ou (Y.C.) et al., 2010**	RARP	Retrospective single-surgeon series (first 100 cases)	Moderate-volume (Taiwan center, single surgeon)	Single surgeon; moderate sample; improvements noted are observational; lacks control or risk adjustment; short follow-up for functional outcomes.

**Ploussard et al., 2010**	RARP	Retrospective single-center series (high-volume lap center adopting RARP)	High-volume (experienced laparoscopic center in France)	Center had significant prior lap experience, potentially biasing a faster LC; no control group; retrospective design.
**Gumus et al., 2011**	RARP	Retrospective single-center series (learning curve analysis)	Moderate-volume (Turkish center, early adoption phase)	Single institution; moderate number of cases; outcomes may be influenced by unmeasured changes in technique; no external validation.
**Ou (Y.C.) et al., 2011a** (Asian J. Surg.)	RARP	Retrospective single-surgeon series (first 60 cases)	Low-volume (same Taiwan surgeon's early cases)	Very limited sample; only initial phase captured; single-surgeon data; statistical power is low to detect plateau.
**Ou (Y.C.) et al., 2011b** (BJU Int.)	RARP	Retrospective single-surgeon analysis (reducing complications over time)	Low-volume (single surgeon, overlapping with above)	Overlaps with surgeon's learning curve data; no independent validation; complication definitions could vary; retrospective.
**Sharma et al., 2010 (pub. 2011)**	RARP	Retrospective single-center report (first 500 cases, 2 surgeons)	High-volume (large UK center, two surgeons in parallel)	Multi-surgeon but single center; learning curve effects may differ between the two surgeons (though combined in analysis); retrospective; no external comparator.
**Lebeau et al., 2011**	RARP	Retrospective single-center study (team-based early learning)	High-volume (experienced team in place)	Single center; early learning period with strong team support (not generalizable to solo surgeons); limited to perioperative outcomes.
**Hashimoto et al., 2013**	RARP	Retrospective single-surgeon series (first 200 cases, Japan)	Moderate-volume (one surgeon's substantial experience)	Single surgeon; no control; possible case selection changes over 200 cases; functional outcomes beyond perioperative period not deeply analyzed.
**Seo et al., 2013**	RARP	Retrospective single-center series (secondary hospital, accumulating experience)	Low-volume (training hospital setting)	Smaller hospital experience; results may reflect resource limitations; retrospective and likely without standardized mentoring, affecting generalizability.

**Vasdev et al., 2014**	RARP	Prospective single-center implementation study (establishing robotic program and fellowship)	Moderate-volume (UK center starting a program)	Uncontrolled observational study; learning curve inferred from timeline; improvements could be due to system/process maturation; moderate sample.
**Davis et al., 2014**	RARP	Retrospective multi-center database study (Premier database, comparing RARP vs open)	High-volume (large national dataset, mixed settings)	Administrative data study; potential coding errors; no randomization – selection bias (cases not randomized to robot vs open); heterogeneity across hospitals (surgeon experience not controlled).
**Di Pierro et al., 2014**	RARP	Retrospective single-surgeon cohort (impact of learning on complications and renal function; n~100)	Moderate-volume (academic center, one surgeon)	Single surgeon's results; specific to patients with extended PLND – may not generalize broadly; retrospective review of complications can miss subtle events.
**Ou (Y.C.) et al., 2014**	RARP	Retrospective single-surgeon series (500 cases, experience in Taiwan)	High-volume (single high-volume surgeon)	One surgeon's very large series; no external validation; improvements over 500 cases could also relate to technology changes over time; retrospective analysis only.
**Good et al., 2015**	RARP	Retrospective comparative study (two experienced surgeons: open/lap vs robotic, matched by volume)	High-volume (two high-volume surgeons, multi-center)	Not randomized (surgeon vs surgeon comparison, inherent differences may exist); limited to two surgeons' experiences; focuses on surgeon skill, may not reflect average learner.
**Sumitomo et al., 2015**	RARP	Retrospective comparative cohort (laparoscopy-naïve center: experienced open vs novice surgeons performing RARP; n≈100)	Low-volume (limited-case center in Japan)	Unique setup but limited generalizability (unusual scenario of no lap experience); small subgroups; outcomes could be confounded by differing surgical skill levels; retrospective.
**Chang et al., 2016**	RARP	Retrospective single-center series (learning curve at a Chinese center; n≈100)	Moderate-volume (single center, initial experience)	Single-center data from early adoption; patient population specifics (China) may

				differ from others; no control; moderate sample size.
**Adili et al., 2017**	RARP	Retrospective single-surgeon series (learning curve of an experienced lap surgeon new to RARP)	Moderate-volume (Canadian academic center starting robotics)	Single surgeon (prior lap expertise may accelerate learning); focuses on margin rates; limited sample during learning phase; retrospective design.
**Fossati et al., 2017**	RARP	Retrospective multi-surgeon analysis (outcomes of 4 high-volume surgeons; focus on continence recovery)	High-volume (experienced surgeons at tertiary centers)	High-volume context – not reflective of typical learning curve for average surgeon; possibly a post-learning curve outcome focus; retrospective.
**Wang et al., 2017**	RARP	Retrospective cohort study (outcomes when introducing a new RARP surgeon into a high-volume team)	High-volume (established high-volume center)	Specific scenario (team dynamics in high-volume group); results may not apply to low-volume settings; not randomized – new surgeon's cases might differ systematically.
**Islamoglu et al., 2018**	RARP	Retrospective single-center study (learning curve effect on positive margins)	Moderate-volume (Turkish center, single surgeon)	Narrow outcome focus (PSM only); may overlook other learning aspects; single surgeon; retrospective.
**Jaulim et al., 2018**	RARP	Retrospective comparison (two surgeons' outcomes over learning curves in a changing referral practice)	Moderate-volume (UK center, evolving case mix)	Two-surgeon comparison without random assignment of cases; referral pattern changes over time confound outcomes; moderate sample; retrospective.
**Monnerat Lott et al., 2018**	RARP	Retrospective multi-surgeon series (surgeons without lap experience learning RARP; Brazil)	Low-volume (several novice surgeons, new program)	Surgeons lacked prior MIS experience, so results represent steep learning but not typical if training exists; small number of cases per surgeon; no uniform training protocol.
**Schiavina et al., 2018**	RARP	Prospective structured training study (impact of intensive modular training on LC)	High-volume (training program in experienced center)	Specific training intervention studied – results applicable only with similar training; no control group (pre/post design); single center.

**Bravi et al., 2019**	RARP	Retrospective multi-institutional study (impact of surgeon experience on margins and BCR; large cohort)	High-volume (pooled data from expert surgeons in multiple centers)	Multi-center retrospective; primarily includes experienced surgeons (learning mostly completed) – more of an outcomes study than LC analysis; potential heterogeneity in techniques across centers.
**Ucar et al., 2019**	RARP	Retrospective single-center study (learning curve effect on surgical, functional, oncologic outcomes in nerve-sparing RARP)	Moderate-volume (single center in Turkey)	All patients underwent nerve-sparing, which may limit generalizability; moderate case number; observational (no control for time effects other than case number).
**Chen et al., 2020**	RARP	Retrospective single-surgeon series (first 500 cases by one surgeon in China)	High-volume (large case series by a single surgeon)	Single surgeon's experience; improvements over 500 cases could include external factors (e.g., technology evolution); no comparative arm; retrospective.
**Song et al., 2020**	RARP	Retrospective cohort (one surgeon as a novice comparing initial RARP vs initial open RP)	Low-volume (single surgeon, limited prior experience in any RP)	Non-randomized comparison of surgical approach (surgeon's learning in both open and robotic concurrently); very specific scenario (novice in both techniques); limited sample in each arm.
**Baunacke et al., 2021**	RARP	Retrospective multi-surgeon study (comparing early vs late outcomes, open vs robotic experience influence)	Moderate-volume (German center(s), mix of experience)	Some surgeons had prior open experience, others learning robotics – heterogeneity; endpoints focus on improvement rate; retrospective data, possibly pooled from multiple surgeons.
**Ambinder et al., 2022**	RARP	Retrospective single-center study (detailed analysis of operative time components over LC)	High-volume (tertiary center, experienced surgeons)	Focuses only on operative time segments; single center with possibly expert bias; does not evaluate oncologic or functional outcomes; retrospective timing data.

**Bock et al., 2022**	RARP	**Prospective** multicenter study (large cohort across several hospitals, LC analysis)	High-volume (multi-institutional, >1,000 cases combined)	No randomization, but as a prospective registry, selection bias is minimized though centers varied; heterogeneity in surgeon skill and technique across sites; endpoints mainly perioperative.
**Gandi et al., 2022**	RARP	Retrospective multi-surgeon study (LC analysis for positive surgical margins overall and by location)	High-volume (single high-volume center with multiple surgeons)	Outcome focused solely on margins; other LC aspects not considered; retrospective review; surgeon technique differences possible even within one center.
**Hashine et al., 2023**	RARP	Retrospective single-center study (institutional learning curve effect on patient-reported functional outcomes)	Moderate-volume (Japanese center, collective experience over time)	Single center, no external validation; improvements in functional outcomes over time could be due to other improvements in care; retrospective; patient-reported outcomes may have response bias.
**Perera et al., 2022**	RARP	Retrospective multi-surgeon series (learning curves and outcomes in an Australian setting)	Moderate-volume (Australian academic center, multiple surgeons)	Moderate sample size; likely variability between surgeons combined in analysis; retrospective and limited by short follow-up for some outcomes.
**Carlos et al., 2024**	RARP	Retrospective multi-surgeon study (trifecta learning curve for surgeons with prior laparoscopy experience)	Moderate-volume (international collaboration or single center in Latin America)	Surgeons were not robotics-naïve (had lap experience), which accelerates learning – results may not apply to truly novice surgeons; observational study without control; moderate sample per surgeon.
**Haseebuddin et al., 2010**	RAPN	Retrospective single-surgeon series (experienced open surgeon's initial robotic partial nephrectomies)	Moderate-volume (large US center, surgeon skilled in open surgery)	Small cohort of initial cases; single surgeon bias; outcomes might be better than a true novice due to prior experience; retrospective, no control.
**Mottrie et al., 2010**	RAPN	Retrospective multi-surgeon series (impact of experience on RAPN outcomes)	High-volume (pioneering robotic center in Europe)	Likely multiple surgeons combined; no clear cutoff for learning plateau defined;

				retrospective; high-volume context not representative of all centers.
**Lavery et al., 2011**	RAPN	Retrospective single-surgeon case series (laparoscopic expert transitioning to RAPN)	High-volume (expert surgeon, high skill environment)	Single surgeon (Palese) with prior extensive lap experience – atypical learning curve; small sample of initial robotic cases; retrospective.
**Pierorazio et al., 2011**	RAPN	Retrospective comparative study (robotic vs laparoscopic partial nephrectomy outcomes and LC)	High-volume (major academic center – Johns Hopkins)	Non-randomized comparison of surgical modality; surgeons had differing baseline skills (robotic technique evolving); retrospective design; potential selection bias of patients for robotic vs lap.
**Tobis et al., 2011**	RAPN	Retrospective single-center series (first 100 RAPN cases)	Moderate-volume (single institution, experienced surgeons in other MIS)	Single center's early experience; no external comparison; learning primarily measured by trends in outcomes over cases – no formal statistical LC analysis; retrospective.
**Yuh et al., 2012**	RAPN	Retrospective single-center study (integration of RAPN into existing robotic program)	High-volume (established robotic program adopting kidney cases)	Context of an existing program means support staff experienced – may shorten learning; data source unclear (possibly conference abstract, limited details); likely retrospective; small sample.
**Dube et al., 2015**	RAPN	Retrospective single-surgeon series (LC effect on warm ischemia time and factors influencing WIT)	Moderate-volume (US high-volume surgeon, new to robot)	Focus on one outcome (WIT); single surgeon (Sundaram) with prior lap experience; retrospective review; small sample of initial cases; no functional/oncologic outcome evaluation.
**Hanzly et al., 2015**	RAPN	Retrospective multi-surgeon comparison (RAPN vs laparoscopic PN learning curves)	Moderate-volume (two centers' experience combined)	Pooled data from different surgeons/institutions; not randomized – case selection for robot vs lap may differ; definitions of LC stages somewhat arbitrary; retrospective.

**Xie et al., 2016**	RAPN	Retrospective single-center study (learning curve vs tumor complexity and outcomes)	Moderate-volume (Chinese center, intermediate case load)	Single center; moderate number of cases, but tumor complexity varied – potential confounder; retrospective design; no external validation; off-clamp vs on-clamp approaches not separately analyzed.
**Dias et al., 2018**	RAPN	Retrospective single-surgeon series (LC impact on perioperative outcomes)	Moderate-volume (Indian tertiary center, initial RAPN adoption)	Single surgeon; relatively small cohort; retrospective; context-specific challenges (resource constraints) not accounted for; outcomes beyond perioperative period not reported.
**Omidele et al., 2018**	RAPN	Retrospective single-center study (trifecta outcomes as metric of RAPN learning curve)	Moderate-volume (US academic center with experienced team)	Single center (Mount Sinai); moderate case number; uses "trifecta" which, while useful, may not capture all learning aspects; retrospective and no comparison to earlier surgical modality.
**Bajalia et al., 2020**	RAPN	Retrospective single-institution study (advanced perioperative outcomes to define true LC)	Moderate-volume (US center, multiple surgeons likely)	Definition of "true" learning curve is post hoc and specific to chosen metrics; data retrospective; potential multiple surgeons with different learning rates pooled together; moderate sample size.
**Castilho et al., 2020**	RAPN	Retrospective single-surgeon series (direct transition from open to robotic PN, aiming for trifecta)	Moderate-volume (Brazil, experienced open surgeon new to RAPN)	Single surgeon; context of prior open experience (results may overestimate performance of a pure novice); relatively small number of cases in analysis; retrospective review.
**Motoyama et al., 2020**	RAPN	Retrospective single-surgeon series (initial RAPN by an experienced robotic surgeon)	High-volume (Japanese center, surgeon experienced in robotic prostatectomy)	Unique scenario – surgeon already skilled in robotics (shorter LC expected); single surgeon; small initial series; retrospective.

**Fiorello et al., 2021**	RAPN	Retrospective single-center comparison (expert vs trainee learning curve in RAPN)	High-volume (Italian center, one expert mentor with trainee team)	Specific to a training setup (expert supervising trainees); not generalizable to settings without proctoring; moderate sample; retrospective analysis of outcomes between two groups without randomization.
**Zeuschner et al., 2020**	RAPN	Retrospective single-center comparative study (10-year, 880 patients: open vs robotic PN outcomes over time)	High-volume (large German center, long-term experience)	Not a pure learning curve study (mixes open and robotic outcomes); possible selection bias in which cases went robotic vs open over the decade; multiple surgeons over time; retrospective.
**Al-Nader et al., 2023**	RAPN	Retrospective single-center study (CUSUM analysis of RAPN learning curve)	High-volume (German high-volume center with experienced surgeons)	While CUSUM provides objective LC analysis, data are retrospective; likely reflects an expert's later cases too, not only initial learning; single center; results might not apply to low-volume settings.
**Zhang et al., 2023**	RAPN	Retrospective single-center study (learning curve for off-clamp RAPN to achieve proficiency)	Moderate-volume (Chinese center, surgeons with some prior experience)	Specific to off-clamp technique (higher complexity, steeper LC); moderate case number; retrospective; findings (e.g., ~18 cases for proficiency) are technique-specific and may differ for standard RAPN.
**Pruthi et al., 2008**	RARC	Retrospective single-surgeon series (initial RARC cases and learning curve evaluation)	Low-volume (early RARC adopter, limited cases at the time)	Very small sample (early feasibility study); outcomes from initial cases only; no comparison to open or later cases; retrospective.
**Guru et al., 2009**	RARC	Retrospective single-center series (learning curve report from initial experience)	Low-volume (early experience at tertiary center)	Small number of cases (pilot experience); performed at a time of limited robotics experience; lacked standardized benchmarks; retrospective.

**Hayn et al., 2010a** (Eur Urol)	RARC	Retrospective multi-institutional series (International Robotic Cystectomy Consortium early results)	High-volume (pooled data from multiple centers)	Multi-center heterogeneity (surgeon skill and patient selection vary); data from early adopters only – results reflect learning phase across institutions; retrospective collection.
**Hayn et al., 2010b** (BJU Int)	RARC	Retrospective single-center series (first 164 consecutive RARCs at one center)	High-volume (Roswell Park, high-case load by an expert team)	Single high-volume center's initial cases – outcomes might be better than average due to center expertise; no control group (e.g., open cystectomy outcomes for comparison in that period); retrospective.
**Richards et al., 2011**	RARC	Retrospective single-surgeon series (first 60 RARC cases at program initiation)	Low-volume (community/academic hybrid setting starting robotics program)	Initial 60-case outcomes likely include learning-related compromises; limited sample; single surgeon; compared to institutional expectations rather than a control group; retrospective.
**Collins et al., 2014**	RARC	Retrospective multi-center study (intracorporeal neobladder outcomes vs learning curve stage)	High-volume (experienced surgeons in Europe implementing intracorporeal diversion)	Focus on intracorporeal diversion subset; multiple surgeons from high-volume centers – not generalizable to low-volume centers or extracorporeal diversion; moderate sample per surgeon; retrospective analysis.
**Desai et al., 2014**	RARC	Retrospective single-center case series (132 intracorporeal neobladders by experienced team)	High-volume (Cleveland Clinic, very experienced surgeons)	No formal learning curve analysis (all cases included, many beyond learning phase); reflects a center of excellence – outcomes likely better than those early in learning elsewhere; retrospective.
**Honore et al., 2019**	RARC	Retrospective single-center series (initial RARC outcomes in an Australian center)	Low-volume (Australian experience, starting phase)	Modest sample size; single center new to RARC (steep learning, outcomes possibly suboptimal initially); no direct comparison to open; retrospective.

**Porreca et al., 2020**	RARC	Retrospective single-center series (learning curve in totally intracorporeal RARC at a high-volume center)	High-volume (Italy, high-volume tertiary center)	Single center of excellence; surgeons already proficient in RARC moving to intracorporeal diversion – learning curve mainly for diversion technique; no control group; retrospective.
**Lombardo et al., 2021**	RARC	Retrospective multi-surgeon study (CUSUM-based learning analysis for intracorporeal RARC, benchmarking outcomes)	High-volume (multi-institution Italian collaboration, expert surgeons)	High-volume context; complex benchmarking (PASADENA criteria) – not applicable to all centers; primarily focused on quality improvement in expert hands; retrospective data aggregation.
**Tuderti et al., 2020**	RARC	Retrospective single-center study (impact of learning curve on intracorporeal neobladder outcomes, with long-term functional results)	High-volume (experienced center in robotic cystectomy)	Single-center results from a center with significant prior robotic experience; not representative of early adopters; no external validation; retrospective.
**López-Molina et al., 2021**	RARC	Retrospective single-center study (learning curve impact on perioperative outcomes for intracorporeal RARC)	Moderate-volume (Spanish center, intermediate experience)	Single institution; moderate number of cases (learning + consolidation phases); outcomes mainly perioperative; retrospective review.
**Wijburg et al., 2022**	RARC	Retrospective multi-center study (EAU Robotic Urology Section analysis of intracorporeal RARC learning curves)	High-volume (pooled European centers' data, multiple surgeons)	Multi-center heterogeneity; surgeons likely already experienced (ERUS working group participants); results show general trends but may not apply to a single low-volume center; retrospective.
**Achermann et al., 2023**	RARC	Retrospective single-surgeon series (learning curve for RARC with intracorporeal ileal conduit by one surgeon)	High-volume setting (experienced open surgeon learning robotics at academic center)	Single surgeon (Walz et al.); surgeon's extensive open experience may shorten robotic LC; focuses on perioperative outcomes; retrospective; limited to ileal conduit diversion.

**Diamand et al., 2023**	RARC	**Prospective** multi-institution pilot study (ERUS robotic cystectomy training curriculum validation)	Moderate-volume (trainee surgeons under proctoring at expert centers)	Small sample (pilot study) of trainees; conducted in ideal conditions (proctors present), which may not reflect unmentored learning; short-term outcomes only (no long-term oncologic data due to pilot nature).
**Tuderti et al., 2024**	RARC	Retrospective single-center long-term study (10-year functional outcomes after intracorporeal "Padua" neobladder, analyzing learning curve effect)	High-volume (single high-volume center with sustained experience)	Single-center, single-technique focus; reflects a matured program's outcomes (learning curve effects mostly already overcome in later cases); not an interventional study – observational trends over a decade; retrospective.
